# Quantitative Structural Description of Zeolites by Machine Learning Analysis of Infrared Spectra

**DOI:** 10.1021/acs.inorgchem.2c04395

**Published:** 2023-04-14

**Authors:** Alina A. Skorynina, Bogdan O. Protsenko, Oleg A. Usoltsev, Sergey A. Guda, Aram L. Bugaev

**Affiliations:** † The Smart Materials Research Institute, Southern Federal University, Sladkova 178/24, 1344090 Rostov-on-Don, Russia; ‡ Paul Scherrer Institute, Forschungsstrasse 111, 5232 Villigen, Switzerland

## Abstract

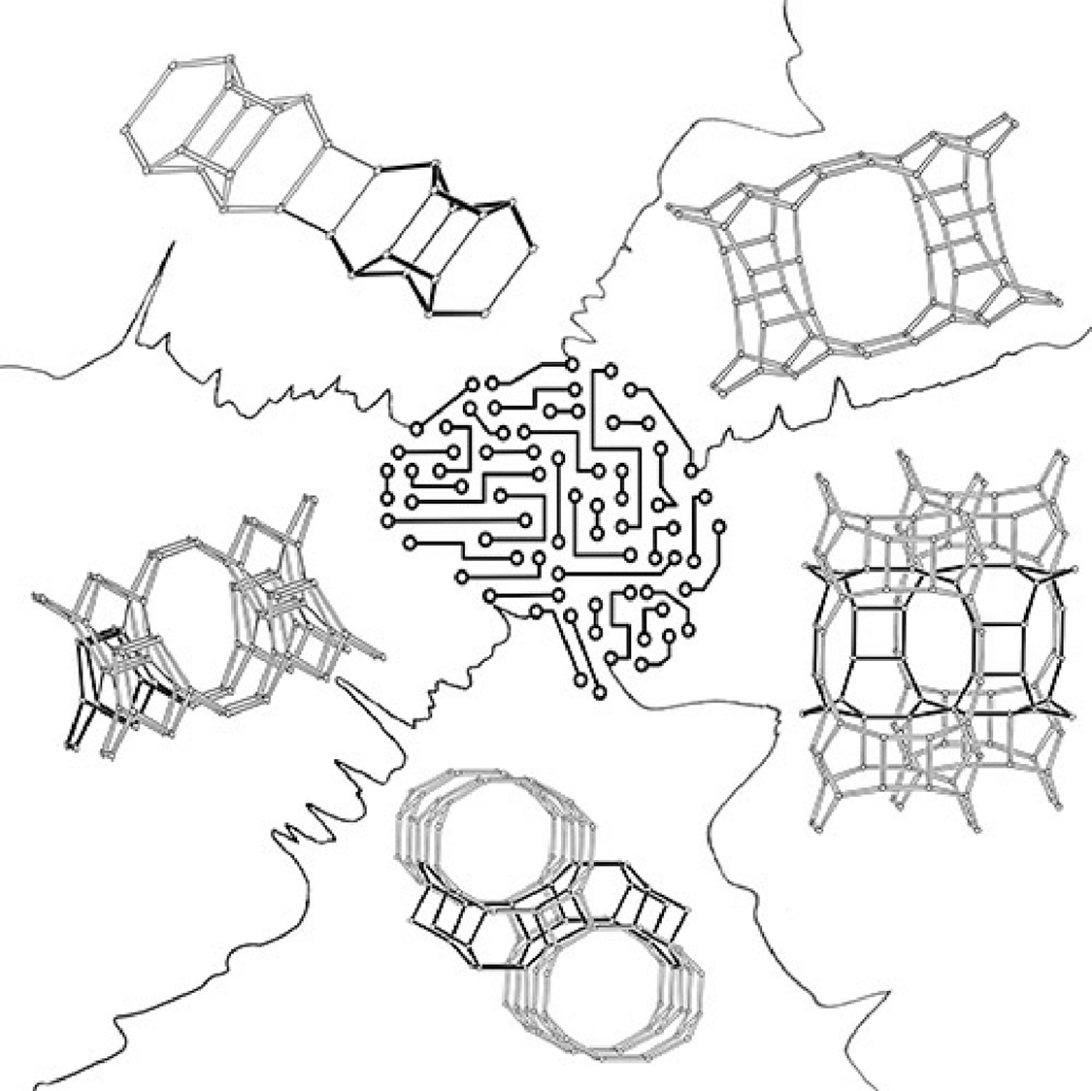

Application of machine learning (ML) algorithms to spectroscopic data has a great potential for obtaining hidden correlations between structural information and spectral features. Here, we apply ML algorithms to theoretically simulated infrared (IR) spectra to establish the structure-spectrum correlations in zeolites. Two hundred thirty different types of zeolite frameworks were considered in the study whose theoretical IR spectra were used as the training ML set. A classification problem was solved to predict the presence or absence of possible tilings and secondary building units (SBUs). Several natural tilings and SBUs were also predicted with an accuracy above 89%. The set of continuous descriptors was also suggested, and the regression problem was also solved using the ExtraTrees algorithm. For the latter problem, additional IR spectra were computed for the structures with artificially modified cell parameters, expanding the database to 470 different spectra of zeolites. The resulting prediction quality above or close to 90% was obtained for the average Si–O distances, Si–O–Si angles, and volume of TO_4_ tetrahedra. The obtained results provide new possibilities for utilization of infrared spectra as a quantitative tool for characterization of zeolites*.*

## Abstract 2

The article provides a detailed study of the machine learning applications for the training set of theoretically calculated infrared spectra for various zeolite structures with the aid of density functional theory. The set of structure-related descriptors was suggested and treated to investigate the ones with the highest prediction quality. It was applied both on the regression approach for the continuous descriptors such as Si−O distances and Si−O−Si angles and classification for natural and secondary building unit prediction.

## Introduction

Zeolites are the class of microporous crystalline materials that are widely used in various industrial,^1,2^ electronic,^3^ medical,^4,5^ agricultural,^6,7^ and other applications. Within each of the known topologies, several structural parameters can be varied such as the Si:Al ratio (SAR) and the type and degree of ion exchange.^8^ To improve the properties and efficiency of the zeolite, new synthesis methods^9^ and post-synthetic treatments are used, leading to the formation of modified zeolites.^10−12^ The most common methods for structural characterization of synthesized zeolites are X-ray diffraction (XRD)^13^ and nuclear magnetic resonance (NMR) spectroscopy.^14^ In many cases, to fully describe the structure, several methods should be applied, which is time- and resource-consuming. This encourages scientists to develop additional approaches within the known methods by changing the measurement procedure itself, writing computer codes to automatically process the data, and combining several methods carried out at the same time in a single analytical software. Furthermore, the application of machine learning (ML) algorithms may allow extracting additional information from experimental data that is inaccessible by conventional approaches.^15^

Vibrational methods, in particular infrared (IR) and Raman spectroscopies, are readily available in most laboratories. Therefore, new approaches for extraction of additional information from such data would be in high demand. For instance, the group of Paolucci et al.^11^ investigated the ion exchange of zeolites using vibrational spectroscopy methods, both experimentally and theoretically. Their research had a more practical application, but they sharply expressed the lack of information extracted from the vibrational spectra. Lansford and Vlachos have recently suggested an ML approach for extracting the microstructure of metal nanoparticles based on the spectra of the absorbed probe molecules.^15^

An extensive search for correlations between the changes in vibrational modes of zeolites and the presence of certain structural features was carried out by several research groups in the second half of the 20th century. Flanigen et al. studied a number of different types of zeolites using experimental IR spectroscopy.^16^ In this article, primary and secondary structural elements, as well as larger symmetrical polyhedra, are identified for each of the zeolites. For many of these elements, they succeeded in matching them with the vibrational modes. In the work by Fichtner-Schmittler et al.,^17^ as well as another one by the group of Beyer et al.,^18^ an attempt was made to correlate the unit cell parameters of a Y-type zeolite with the stretching modes of the IR spectra and the aluminum content in the system. As a result, linear dependencies of wavenumbers on T–O bonds and O–T–O angles were obtained. It should be noted that the described results revealed some special cases, and up to date, there is no structured database of calculated vibrational modes for structures of zeolite frameworks, and the present work is aimed at closing this gap. More recently, ML algorithms were also applied for classification task using Delaunay tessellation as a tool of classification^19^ and with the smooth overlap of atomic position (SOAP) method.^20^

There are different ways of zeolite classification by their component units. Primary building units are single TO_4_ tetrahedra, where the T-atoms or the T-sites are tetrahedrally coordinated Si or Al atoms. In 2007, Van Koningsveld^21^ presented an extensive list of 58 composite building units (CBUs) and five chains that are found in at least two different framework types. On the other hand, there are secondary building units (SBUs), which are derived from the assumption that the entire framework consists of only one type of SBU. In contrast to CBU, SBUs are always non-chiral. Another way of zeolite framework representation is the natural building units (NBU) or the natural tilings suggested by Proserpio et al.^22,23^ This classification has several advantages. First, the choice of NBU is unambiguous and can be easily carried out by computer methods. Second, NBUs completely compose the crystal space of the framework. Third, natural tiles enumerate all minimal cavities in the framework; all larger cages can be obtained by gluing the tiles. The last two approaches described above will be taken into account in this work. A very extensive review on the other methods for classifying zeolite structures has been considered by Armbruster and Gunter^24^ in 2001. However, classification of the present study will not include additional parameters not available in the database of the International Zeolite Association (IZA),^25^ and the main focus of the work will be concentrated on the regression problem of finding the dependences of vibrational modes and structural parameters.

Here, we report on the application of ML algorithms to a vast database of the simulated IR spectra of 230 different types of zeolites to predict the presence or absence of the structural units available in the database of the International Zeolite Association and determine continuous parameters, among which, based on the ideas suggested in refs (26) and (27), we considered different structural units from Si–O interatomic distances and Si–O–Si angles to the volumes of TO_4_ tetrahedra, cell volumes, and densities.

## Methods

### Computational Details

Generally, zeolites contain silicon, aluminum, and oxygen in their framework and cations, water, or other light molecules within their pores. However, for the present study, the frameworks were simplified and represented only by silicon and oxygen atoms in the framework without the addition of cations and adsorbed molecules. Such an approach with only silica zeolites is not the first time to be used in modeling zeolites.^28−31^

The unit cells for 230 zeolite frameworks were taken from cif files of the IZA database and then simplified to primitive cells. Relaxation for most primitive cells was done in the VASP 5.3^32^ code and included five steps (unless otherwise specified): sequential optimization of ion positions–cell volume–ion positions–cell volume–ion positions. The evolution of the total energy for the computational cell after each step is shown in Figure S1.

For calculations of vibrational spectra, only the Γ-point was considered. Periodic density functional theory (DFT) calculations including geometry optimization and computation of vibrational spectra, obtained from density functional perturbation theory, were performed with the PBE (Perdew, Burke, and Ernzerhof) exchange-correlation^33^ functional. The projected-augmented wave (PAW) method^34^ was used to describe core–electron interactions, and the plane wave basis set was limited to a cutoff energy of 500 eV (Figure S2). The convergence criteria for the electronic self-consistent field loop were set to 10^–6^ and 10^–5^ eV for geometry relaxation.

The selected theoretical vibrational IR modes were compared with the experimental ones (Figures S3–S6 and Table S1) from the RRUFF database^35^ to estimate the accuracy of the approach.

Moreover, the 30 primitive cells from the optimized database were distorted, i.e., uniformly stretched or compressed in three directions up to 4% in 1% increments. For all of these distorted frameworks, only the ion positions were relaxed using the same functional and PAW method as described above. Vibrational spectra were calculated and considered at the database for ML.

All calculations and further analysis were performed automatically using the self-written Python code based on Atomic Simulation Environment (ASE)^36^ and Python Materials Genomics (Pymatgen)^37^ libraries.

### Machine Learning Algorithm

The spectral database was constructed with simulated IR spectra convoluted by the Gaussian function with σ equal to 50, chosen according to experimental peak deconvolution (Figure S7) and then normalized by area. The spectra were taken in a 0–1600 cm^–1^ range with a step of 1 cm^–1^. These spectra were included in the training set for ML. A set of structural descriptors was suggested for both the classification and regression approaches. To solve the classification problem, all natural tiles with secondary building units were used. The list of regression descriptors was as follows: cell parameters and cell volume, number of T-sites and their average volume, four T–O distances and four T–O–T angles for one of the tetrahedra, and their average values for the entire cell, scaling factor of the lattice, and framework density (FD), calculated as the number of T-sites per 1000 Å^3^.

The structures of zeolites were classified using the ExtraTreesClassifier approach. The results of the statistical classification were presented as confusion matrices, also known as error matrices, where each row of the matrix represents the predicted classification label, and each column represents the actual class. The matrix elements were colored according to their values on the color scale on the right. Here, the problem of binary classification was solved. For each framework, class 1 or class 0 was assigned to every possible unit, where the first means the presence of a unit in the structure, and 0 means its absence. The resulting classes were highly unbalanced; thus, the adjusted (normalized) balanced accuracy score was used to calculate the classification accuracy.^38^ Adjusted (normalized) balanced accuracies were zero for random predictions and one for perfect classification.

For regression analysis, the ExtraTreesRegressor from the scikit-learn library^39^ was selected, which showed one of the best *R*^2^-scores for predicting the average Si–O–Si angle (Figures S8 and S9). The PyFitIt^40−42^ code extended for vibrational spectroscopies helps to automate the spectral analysis. To estimate the regression quality, the 10-fold cross-validation was used. The dataset was divided into 10 parts, and then in turn, each part was treated as a validation set and all the others as the training sets. We fitted ExtraTreesRegressor using the training set and made prediction for validation. All the obtained predictions were plotted on the prediction against the true parity scatter plot (see the Results and Discussion section), and the *R*^2^-score was calculated.

Part of the spectra calculated for the distorted frameworks was used as a test set, which allowed us to evaluate the prediction quality based on both the cross-validation (CV) procedure over the whole dataset and the prediction accuracy for the test set.

## Results and Discussion

### Classification Problem

Simulation of IR spectra was performed for 230 various types of zeolite frameworks from the IZA database (Figure S10a) and complemented with additional 240 spectra for the selected 30 types of frameworks with artificially modified cell parameters (Figure S10b). The brightest modes for all presented structures lie in the range of 1400–800 cm^–1^.

**Figure 1:**
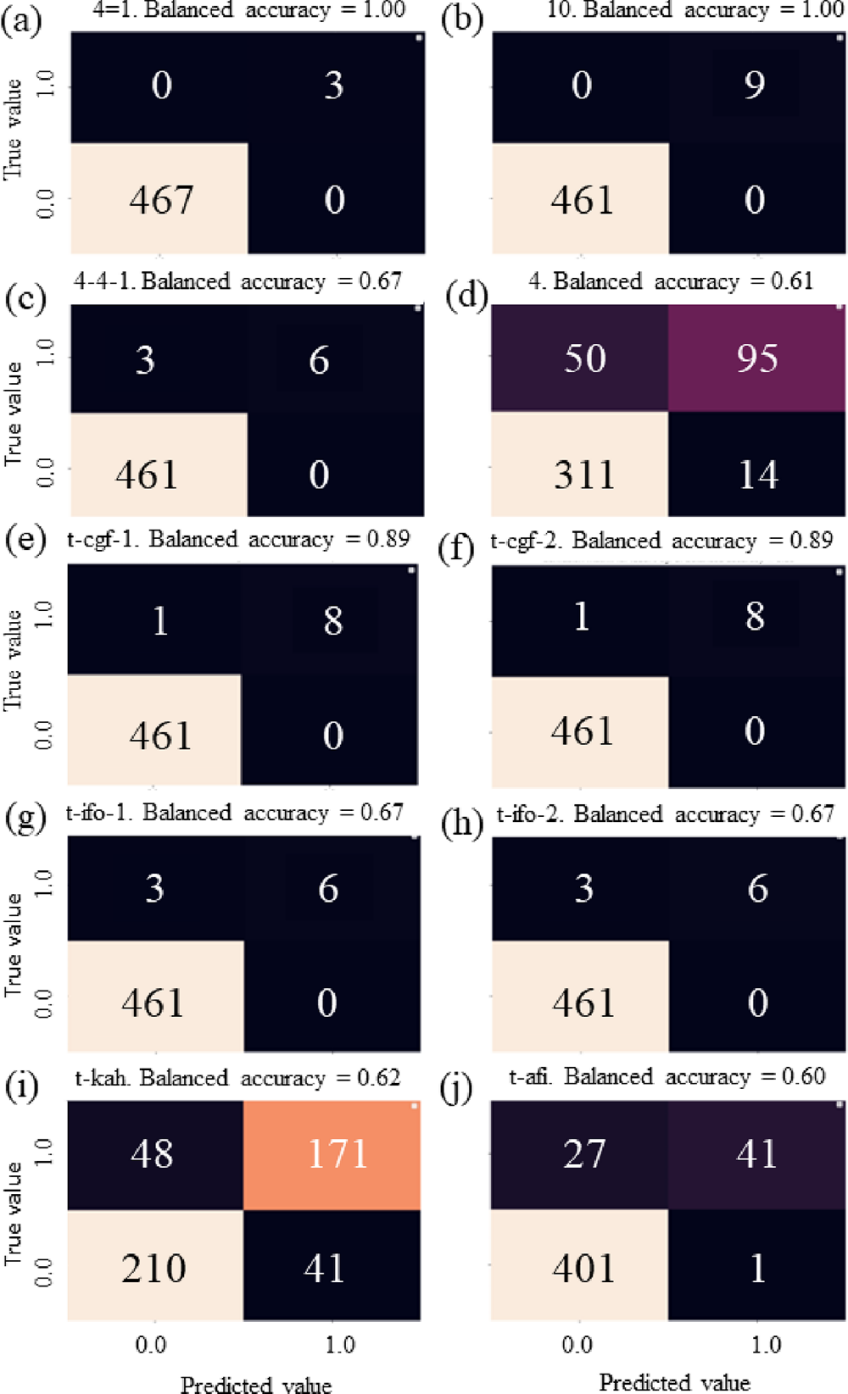
Confusion matrices in solving the classification problem for SBUs 4 = 1 (a), 10 (b), 4-4-1 (c), and 4 (d) and NBUs t-cgf-1 (e), t-cgf-2 (f), t-ifo-1 (g), t-igo-2 (h), t-kah (i), and t-afi (j).

The training set for the classification task included only 230 simulated IR spectra of various types, calculated for the relaxed geometries of zeolites at the PBE level of theory. This set will be referred to as the initial dataset. Each spectrum of the dataset was labeled by a set of structural descriptors: tiles and SBUs present in the structure as well as the space group and the number of Si atoms per unit cell. Categorical structural descriptors were one-hot encoded, and space groups were denoted with a number index. The ExtraTreesClassifier was used to predict each structural descriptor independently from the vibrational spectrum. It was found that none of the NBUs and SBUs give an adjusted (normalized) balanced accuracy above 50%. Then, it was decided to use the entire set of calculated IR spectra, including the initial dataset and 240 spectra for 30 stretched zeolites. Such a dataset was called full. Only four SBUs (4 = 1, 10, 4-4-1, and 4) and six tiles (t-cgf-1, t-cgf-2, t-ifo-1, t-ifo-2, t-kah, and t-afi) (Figure S11) can be predicted with an accuracy higher than 60% (Figure 1).

Thus, the classification algorithm allowed us to predict four tiles and six SBUs with average adjusted (normalized) balanced accuracies of 0.72 and 0.82, respectively. Further development of the classification can be done via Delaunay tessellation^19^ or graph theory,^43^ which help to represent the pores of the zeolite frameworks, which naturally have a significant weight in the structure of the zeolite and are responsible for its main properties.

### Regression Problem

**Figure 2:**
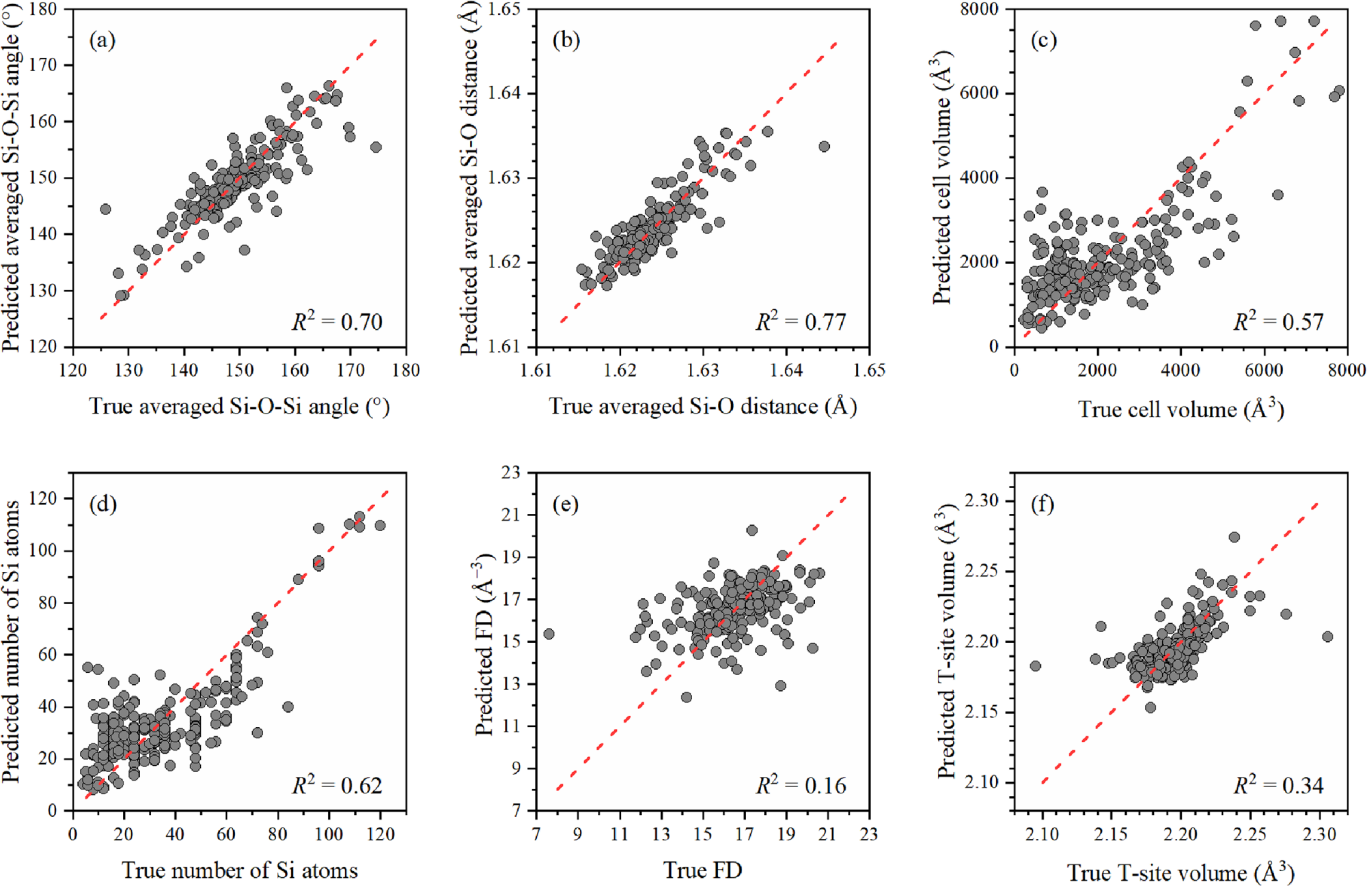
Parity plot of the values, predicted by the ExtraTrees model, against the true ones (circles) for the average Si–O–Si angles (a), average Si–O distances (b), computational cell volume (c), number of Si atoms (d), FD (e), and tetrahedron volume (f) together with perfect model performance (dashed line).

As the next step, a set of continuous structural descriptors was introduced, which included cell parameters and cell volume, number of T-sites and their volume, individual and averaged Si–O distances and Si–O–Si angles, and framework density (FD). As in the case of the classification problem, the 10-fold CV was used. For the following descriptors, good *R*^2^-scores were obtained: average Si–O–Si angles (Figure 2a), average Si–O distances (Figure 2b), volume of the calculated zeolite cell (Figure 2c), number of Si atoms (Figure 2d), framework density (Figure 2e), and tetrahedron volume (Figure 2f). In this case, the best *R*^2^-scores were shown by the average Si–O interatomic distances (*R*^2^-score = 0.77) and the average Si–O–Si angles (*R*^2^-score = 0.70). The reported results were obtained for the initial dataset by using ExtraTreesRegressor, which was chosen by comparing the prediction quality of several ML methods (Figure S7).

Despite the highest *R*^2^-score of the ExtraTrees regressor relative to other ML methods (Figure S9), the absolute value of the *R*^2^-score is still not high enough even for the averaged Si–O–Si angles. The results of ML prediction can often be improved by increasing the training set size. Due to the limited number of available zeolite structures, to expand the training database, the calculation of IR spectra was performed on the artificially deformed zeolite geometries. This complemented the database with additional 240 theoretical IR spectra of deformed structures. It should be noted that if we use the base of unstretched structures as a training sample and predict the descriptors of the stretched base, then only two descriptors will give a positive prediction quality: the volume of the tetrahedron with *R*^2^-score = 0.29 (Figure S12a) and the average Si–O distances with *R*^2^-score = 0.27 (Figure S12b). However, for both descriptors, the prediction quality is even lower than 0.5, which indicates the impossibility of an accurate prediction. This result is explained by the fact that for many deformed zeolites, the values of the structural descriptors are beyond the region of the ones from the initial training database.

Before simple amalgamation of the spectra of distorted zeolites with the initial database, which was expected to improve the prediction quality, it was decided to add only a part of the spectra of the distorted zeolites to the training set (which will be referred to as a mixed dataset), while the rest were included in the test set.

The quality of the prediction for the test set improved greatly compared to the CV results of the training set of undistorted zeolites (Figure S13). In this way, the average Si–O distances were predicted with the accuracy of *R*^2^-score = 0.94 (Table 1) and the average Si–O–Si angles with the accuracy of *R*^2^-score = 0.48. Since the volume of the tetrahedron correlates with the Si–O distances and the Si–O–Si angles, its prediction was also improved significantly to *R*^2^-score = 0.80. The framework density prediction also increased to *R*^2^-score = 0.26. The volume of the computational cell, on the contrary, was no longer predicted, yielding *R*^2^-score = 0.00 (Figure S14a).

**Table 1:** *R*^2^-Scores Obtained by Training ExtraTreesRegressor on Different Spectral Datasets

descriptor of the structure	initial database (10-fold CV for 230 spectra of relaxed zeolites)	mixed database(300 spectra of relaxed and distorted structures in the training set and another 170 spectra for distorted zeolites in the test set)	full database(10-fold CV for 470 simulated relaxed and distorted structures in the training set)	non-convoluted database (same as the full dataset)
average Si–O distances	0.77	0.94	0.94	0.93
average Si–O–Si angles	0.70	0.48	0.80	0.43
TO_4_ volume	0.34	0.80	0.91	0.88
FD	0.16	0.26	0.56	0.37
cell volume	0.57	0.00	0.65	0.73

**Figure 3:**
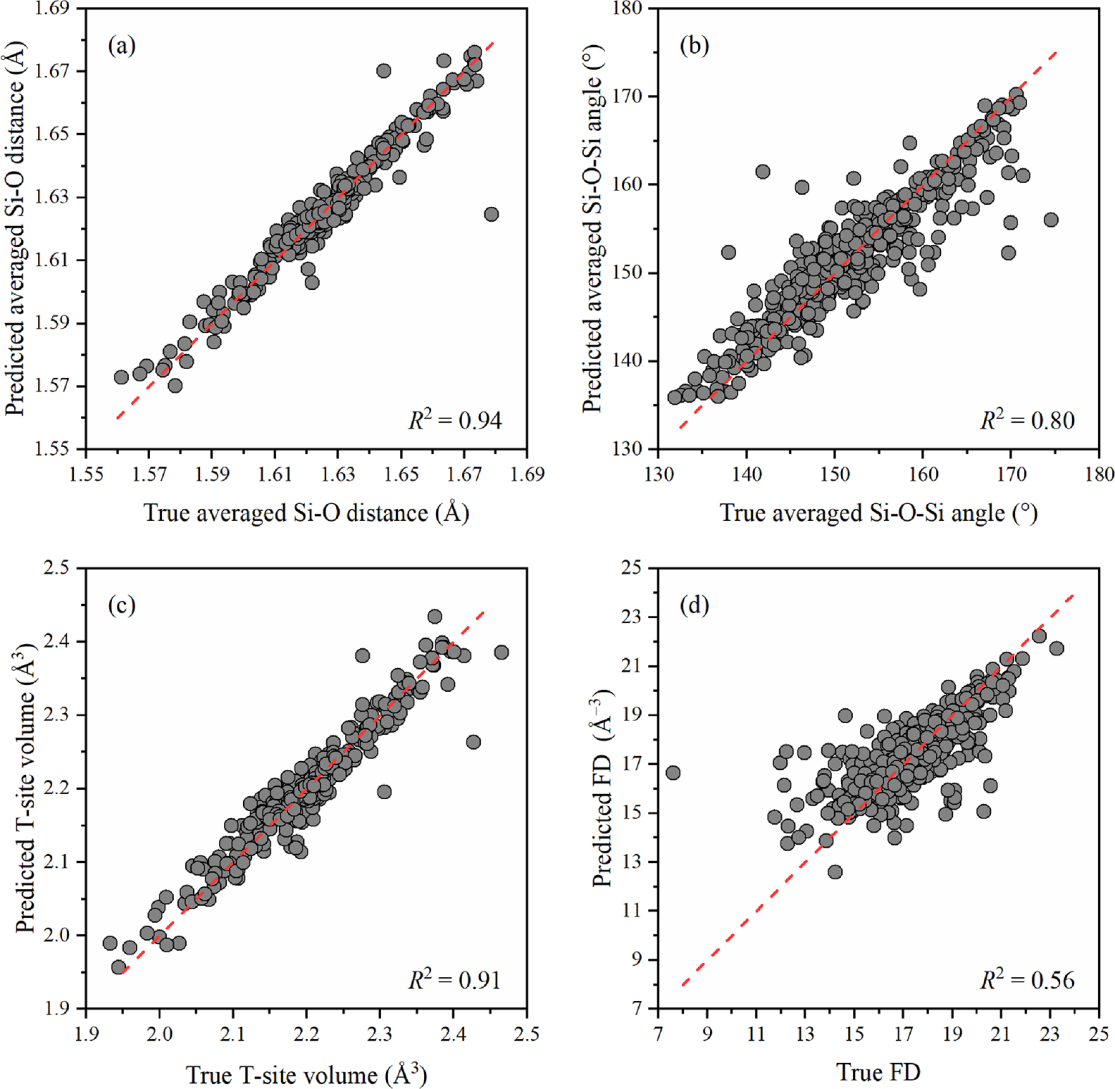
Parity plot of predicted by the ExtraTrees model against true values (circles) with perfect model performance (dashed line) for average Si–O distances (a), average Si–O–Si angles (b), tetrahedron volume (c), and FD (d) for the extended full database of 470 simulated spectra.

Adding all the simulated spectra to one database made it possible to obtain a training set of 470 spectra, called the full set, which significantly improved the prediction quality for all descriptors (see Table 1 and Figure 3). The average Si–O interatomic distances were predicted with an *R*^2^-score of 0.94, the average Si–O–Si angles got an *R*^2^-score of 0.80, and the volume of the tetrahedron got an *R*^2^-score of 0.91. The prediction of FD also improved (*R*^2^-score = 0.56). Cell volume was also predicted with a much higher accuracy (*R*^2^-score = 0.65; Figure S14b); however, the results described above indicate that this descriptor can be poorly predicted if this model is applied to the spectra of zeolites that are not included in the training set.

It is important to note that some of the zeolites have structural parameters significantly different from the rest of the parameter set. In this case, expanding the database near the outlier value will further improve the prediction.

### Spectral Descriptors

To establish the relationship between the structural parameters of the zeolites and the features of their IR spectra and to improve the performance of the ML algorithms by using only informative spectral descriptors, a number of techniques were applied. First, concentration profiles from the multivariate curve resolution alternating least-squares method and projections of the first three to five principal components were used as IR features with the result of significantly lower prediction quality. Then, more intuitive descriptors were utilized. Recently, we successfully applied such an approach to reduce the dimensionality problem in ML application to X-ray absorption spectra,^44,45^ where such descriptors as peak positions and their intensities and curvatures were used. For the current problem, we constructed the non-convoluted dataset based on the full database, where instead of convoluted spectra, only a set of 36 frequencies of the most intensive vibrational modes was used. This number was chosen since it was the minimal number of frequencies among the considered structures. For every structure, the frequencies were sorted according to the corresponding intensity and the 36 most intense ones were taken. Both regression (Table 1 and Figure S15) and classification (Figure S16) tasks were solved for this database. The prediction of Si–O distances and TO_4_ volumes was comparable with the case when the full spectra were used for training. The prediction of the cell volume was improved. At the same time, worse prediction accuracy was observed for Si–O–Si angles and FD. SBUs and tilings are predicted with slightly lower quality than in the case of full spectra. In summary, such a choice of descriptors allowed us to reduce the dimensionality and achieve similar prediction quality for some of the parameters. However, it should be noted that the determination of such descriptors for experimental spectra is not straightforward since it would require the deconvolution procedure to be applied.

**Figure 4:**
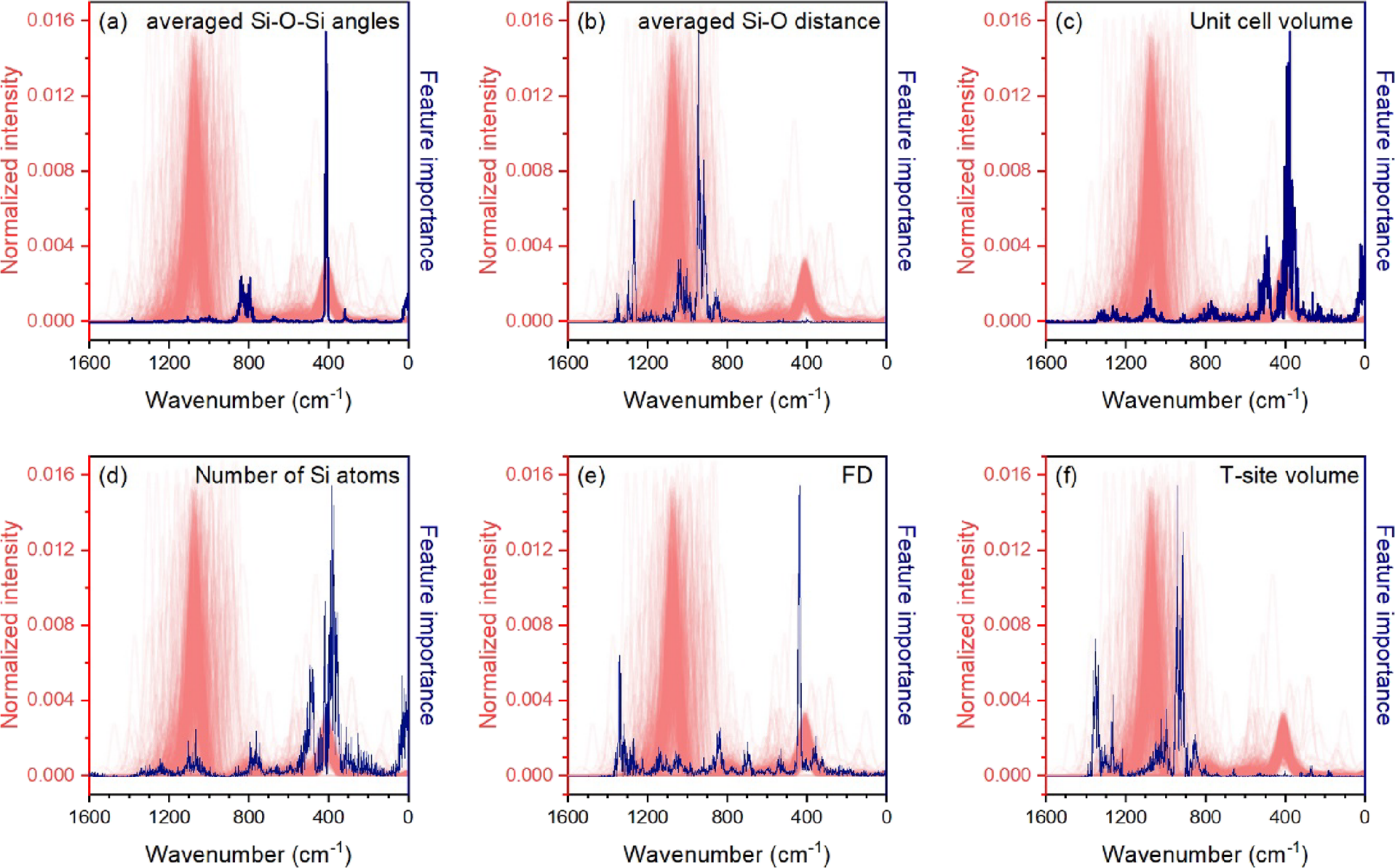
Feature importance for average Si–O–Si angles (a), average Si–O distances (b), computational cell volume (c), number of Si atoms (d), FD (e), and tetrahedron volume (f) constructed for the extended database of 470 simulated spectra.

**Figure 5:**
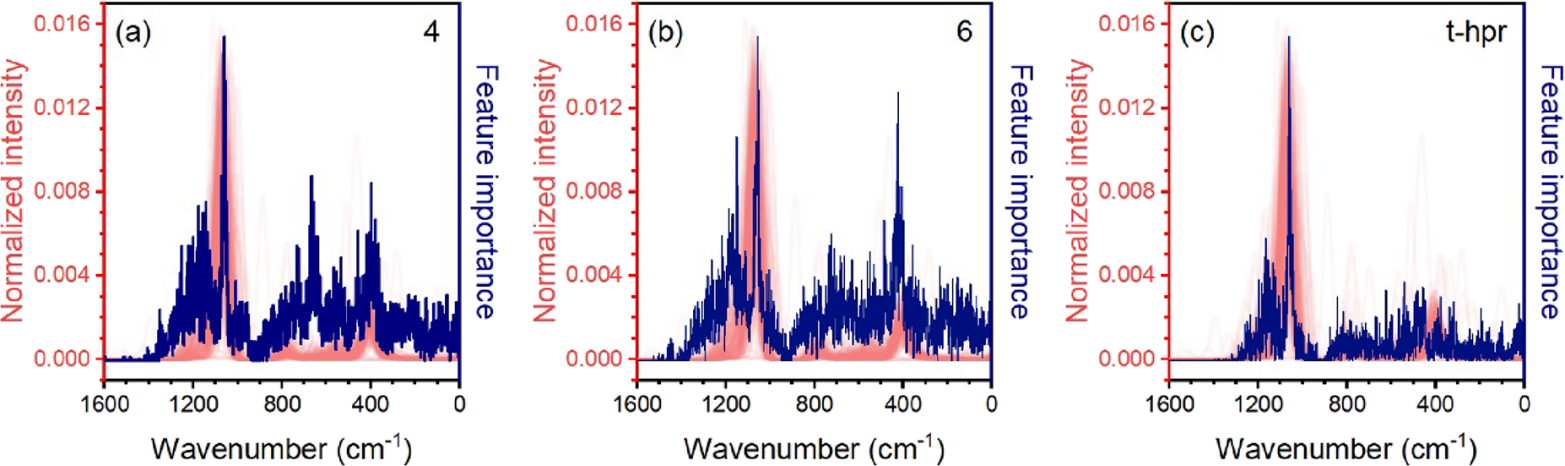
Feature importance for the average 4- (a) and the 6-membered rings (b) and double 6-membered ring as t-hpr tiles (c).

As a next step, to understand which spectral features are required for the ML algorithm for prediction of the structure, the impurity-based feature importance plots were computed as implemented in the scikit-learn library.^39^ Feature importance evaluates all spectrum points for a given descriptor. A higher value of importance means that a given point (region) of the spectrum has a greater influence on predicting a particular descriptor. The result of this evaluation (Figure 4) highlights the importance of the 600–300 cm^–1^ region in the definition of Si–O–Si angles and framework density, which correlates with the number of T-atoms and cell volume, while the TO_4_ volume and interatomic distances are determined from the 1400–800 cm^–1^ range. These results are consistent with the previous assignments made for some of the structural parameters on the basis of a relatively small amount of the experimental infrared spectra.^16^ It can be noted that feature importance plots for models predicting local structural features (Si–O distance and Si–O–Si angle) show a favor to the different parts of the IR spectrum compared with one of the models predicting topological features, such as FD and the number of Si atoms per cell. T-site volume. The vibrational frequencies higher than 800 cm^–1^ appear due to the tetrahedral asymmetric stretching (T-site volume) and asymmetric stretching of some linkages between tetrahedra such as 4- and 6-membered ring SBUs and double 6-membered ring as t-hpr tiles (Figure 5). The feature importance plots for classification task on convoluted spectra show no specific regions, meaning that the determination of tiles and SBUs by ML tends to be a fingerprint-matching of the entire IR spectrum. The specific regions contributing to the prediction of certain structural features suggest a possible further development of the approach toward utilization of a limited set of spectral descriptors instead of the full IR spectra. On the other hand, the fact that simple peak positions and intensities do not provide good prediction while utilization of the full spectrum does highlights the advantages of the ML approach capable of establishing hidden correlations with respect to the classical search of such correlations reported previously^16−18,26^ and valid only for the limited subsets of structures.

### Validation on the Experimental Spectra

Finally, an attempt was made to predict the structure parameters based on 14 experimental FTIR spectra from the RRUFF database by training the model on the full theoretical dataset. However, it has to be taken into account that the theoretical and experimental vibrational modes are shifted relative to each other (see Figure S3). In addition, the width of the peaks did not match, and doubling of some peaks, observed for several experimental spectra, was not reproduced in simulations. This can originate from (i) the principle limitation of the used approach; (ii) differences in experimental and theoretical structures (bond lengths, cell parameters, etc.); and finally, the presence of (iii) Al-sites and (iv) different cations in the experimental samples, which were ignored in the theoretical model.

To account for such differences, the prediction was carried out on a theoretical dataset, for which convolution was varied in the range of peak width (sigma, used for convolution) of 1–100 cm^–1^, with a simultaneous shift of the wavenumber within ±100 cm^–1^ or scaling the whole wavenumber range within ±9% (Figure S7). The prediction of Si–O distances was possible over a wide range of varying parameters, and the best *R*^2^-score was 53% (Table S2). The highest *R*^2^-score prediction of TO_4_ volume was 42%. Other structural parameters were predicted with minor accuracy for the available set of experimental spectra.

In addition, a 10-fold CV procedure for the regression problem was applied to the training set constructed only by experimental spectra (Figure S17). The average Si–O interatomic distances were predicted with *R*^2^-score = 0.58, slightly worse predicted were the average Si–O–Si angles and FD with *R*^2^-scores = 0.39 and 0.36, respectively, and the T-site volume showed only *R*^2^-score = 0.11. Although only 14 experimental spectra were used in the training set, such an approach demonstrates a potential of its applicability to experimental spectra and could be significantly improved by extending the training database. In addition, since the systematic discrepancy between theoretical and experimental spectra was observed, one should consider that the correlation observed based on theoretical simulations may not be observed or may be different in the experimental ones.

## Conclusions

In this work, an advanced tool was proposed for determining the structural features of zeolite frameworks from IR spectra using ML algorithms.

We have built a database of 470 simulated IR spectra to establish structure-vibrational mode relationships for 230 different types of zeolite frameworks based on their topology, interatomic distances, angles, tetrahedron volumes, and framework density. We applied both regression (for continuous structure descriptors, such as framework density, T-site volume, averaged Si–O distances, averaged Si–O–Si angles, and others) and classification (for discreet descriptors, such as the presence of SBU and different membered rings) approaches. The statistical results of the classification problem showed a prediction of several secondary and natural building units with an accuracy greater than 80%.

The regression problem was solved for continuous parameters and showed very promising results in the prediction of Si–O distances, Si–O–Si angles, tetrahedron volume, and framework density, where the extension of the data by stretched frameworks significantly improved the quality of prediction.

Application of ML algorithms to experimental spectra based on the developed theoretical dataset was also investigated. Despite the difference between the theory and experiment, the proposed ML model made it possible to predict the experimental Si–O distances and T-site volume with an *R*^2^-score higher than 42% using theoretical spectra as a training set.

The main difference of this study from previous searches for correlations between spectral data and structural features is the fact that a wide range of zeolites was considered, consisting of 230 different frameworks with their uniformly calculated theoretical spectra. The proposed solution to the classification problem makes it possible to determine the presence of structural subunits only from the vibrational spectrum. The solution of regression problems provides the relationship of certain structural features and vibrational modes in the frequency range of 1400–200 cm^–1^. The established correlations can be especially useful to monitor *in situ* the evolution of zeolite’s frameworks under external conditions (temperature, pressure, and reactive substrate) by IR spectroscopy.

## Supporting Information Available

Details of the ML algorithms, structural details, additional results of regression, and classification problems (PDF)
