# Supplementary material for: Quantitative Structural Description of Zeolites by Machine Learning Analysis of Infrared Spectra
Source: Inorg Chem. 2023 Apr 14;62(17):6608–16. doi: 10.1021/acs.inorgchem.2c04395 (PMC10155178; doi:10.1021/acs.inorgchem.2c04395)
Supplement: Supplementary file 1 — ic2c04395_si_001.pdf [file ic2c04395_si_001.pdf]

Supporting information for:

QUANTITATIVE STRUCTURAL DESCRIPTION OF ZEOLITES BY MACHINE-LEARNING ANALYSIS OF INFRARED SPECTRA

Alina A. Skorynina,[1*] Bogdan O. Protsenko,[1] Oleg A. Usoltsev,[1] Sergey A. Guda,[1] Aram L. Bugaev[2*]

[1]The Smart Materials Research Institute, Southern Federal University, Sladkova 178/24, 344090, Rostov-on-Don, Russia

[2]Paul Scherrer Institute, Forschungsstrasse 111, 5232, Villigen, Switzerland

*Corresponding author: alinaskorynina@gmail.com, aram.bugaev@psi.ch

The optimization of framework structures was carried out in 5 steps. An example of how the total energy changes with each step is shown in Figure S1 for three frameworks with different volumes of the primitive cell used for the calculation. The cell volume of EDI is 313.28 Å^3, GME – 1613.85 Å^3, and KFI – 3252.32 Å^3. For larger structures, after the third optimization step, there are still changes in the total energy in the second decimal.

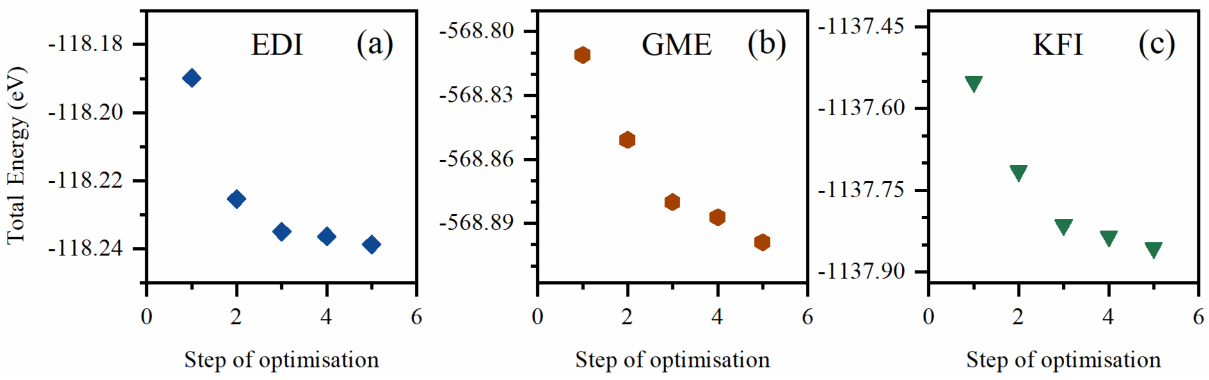

Fig. S1. Evolution of the total energy the ion-electron system vs. step of optimization for zeolite frameworks EDI (a), GME (b) and KFI (c).

The plane wave basis set size in VASP is determined by the minimum value of the cut-off energy (ENCUT) above which the total energy of the entire system does not change or changes insignificantly. Figure S2 shows the dependence of the system energy on the ENCUT parameter in the range from 250 to 1000 eV for the same frameworks. It was decided to use an ENCUT value of 500 eV that is optimal in terms of the time required to perform the calculations. This value was used for all structures in the present study.

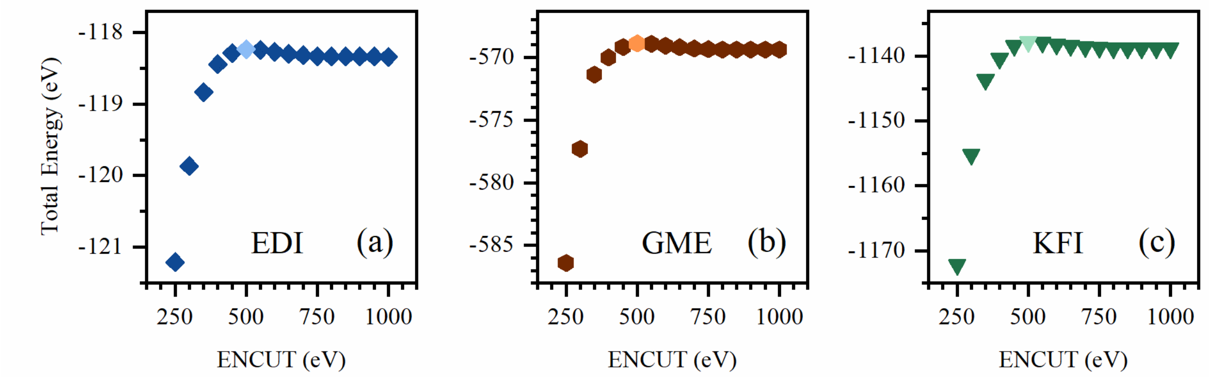

Fig. S2. The dependence of the total energy the ion-electron system on the plane wave basis set for EDI (a), GME (b) and KFI (c). The selected value is highlighted in a lighter shade.

In order to validate the DFT method applied in the manuscript, we plot together the FTIR spectra of natural zeolite minerals, taken from the RRUFF database,[1] and the simulated IR spectra for the same topologies containing only silicon and oxygen atoms (Figure S3). The simulated spectra presented were not shifted relative to the experimental ones. However, it can be noted that many vibrational modes are present in both spectra. Except the shift, the observed difference is due to the obligatory presence of aluminium and other cations in natural minerals, the vibrational modes of which also belong to the region below 1800 cm^{-1}. Figure S4 also shows a comparison of the FTIR spectra of two of the minerals and the simulated spectra of corresponding stretched frameworks.

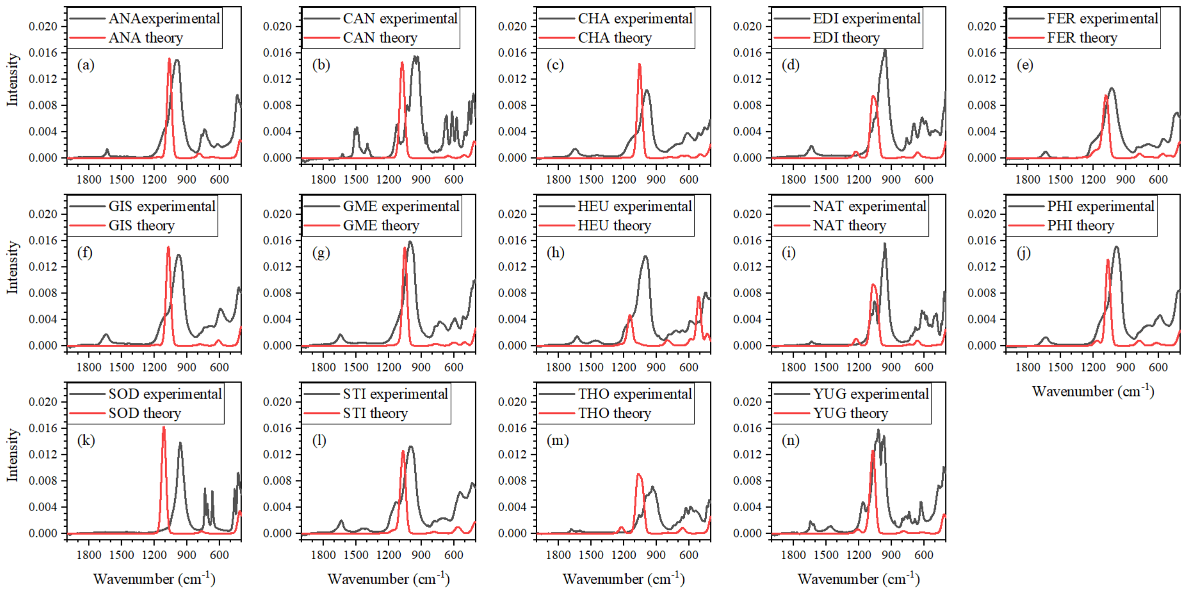

Fig. S3. Comparison of experimental FTIR spectra (black) for natural zeolite minerals (a-n) and simulated IR spectra (red).

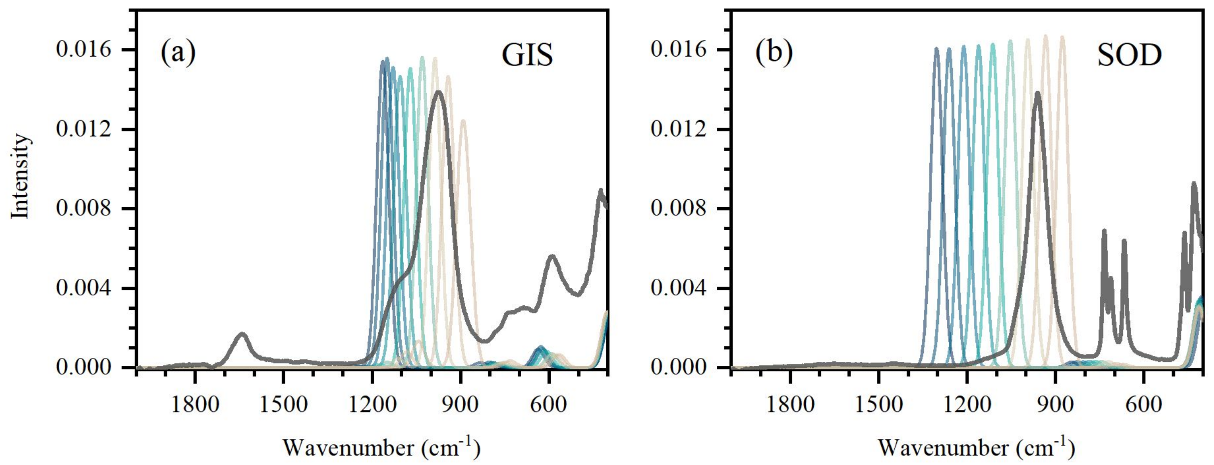

Fig. S4. Comparison of experimental FTIR spectra (black) for the natural zeolite minerals Garronite-Ca (a) and Sodalite (b) with simulated IR spectra of the same topology with distorted cells (from – 4 %, dark blue, to + 4 %, light brown).

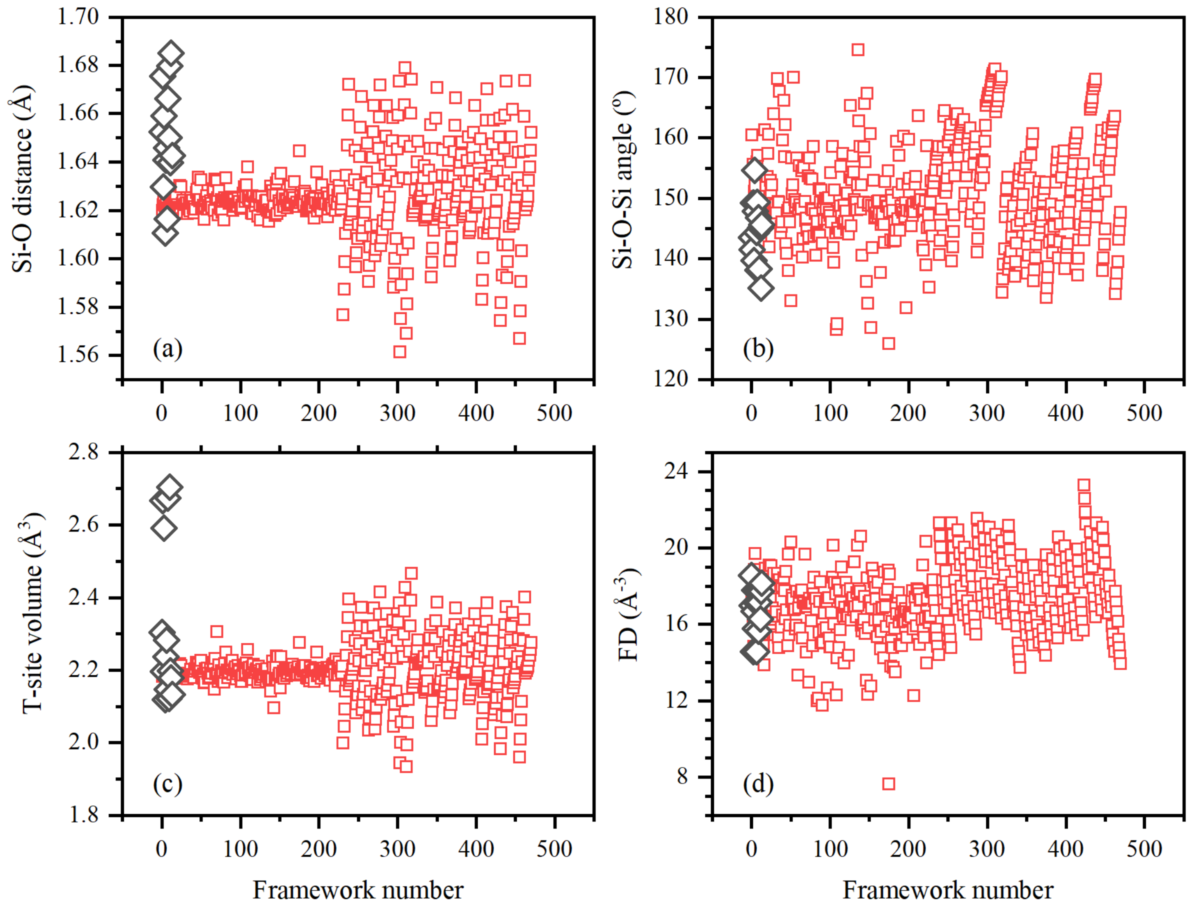

Fig. S5. Representation of structural parameters: Si-O distances (a), Si-O-Si angles (b), TO_4 volumes (c) and FD (d) of natural zeolites (black diamonds) extracted from the references to the RRUFF database, and parameters for the *full* theoretical database (red squares).

Detailed information about the minerals, their chemical composition, the instrumentation setting on which the FTIR spectra were collected, as well as references to the source with data process can be found in the RRUFF database by identification number (ID), the correspondence of which with the mineral is given in the Table S1. Some of the structural parameters for these minerals are plotted in Figure S5 with corresponding parameters present in the theoretical *full* database. The change in the volume of the calculated primitive cells is shown in Figure S6.

Table S1. The list of natural zeolites the FTIR spectra of which were taken from RRUFF database.

Framework name	Mineral name	RRUFF ID
ANA	Analcime	R040128
CAN	Cancrinite	R050352
CHA	Chabazite-Ca	R050014
EDI	Edingtonite	R040110
FER	Ferrierite-Mg	R070091
GIS	Garronite-Ca	R050281
GME	Gmelinite-Na	R050371
HEU	Heulandite-Ca	R040021
NAT	Natrolite	R040102
PHI	Phillipsite-Ca	R050078
SOD	Sodalite	R040141
STI	Stilbite-Ca	R050012
THO	Thomsonite-Ca	R050091
YUG	Yugawaralite	R050407

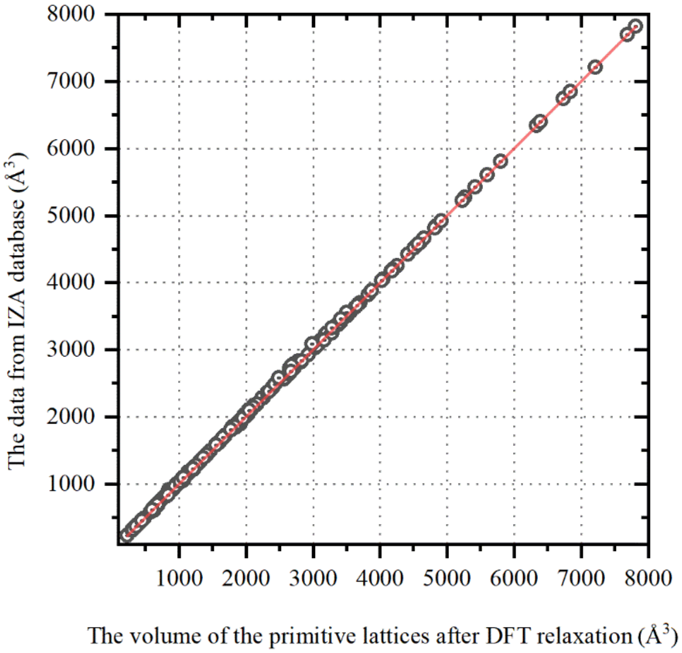

Fig. S6. The volumes of the primitive cells after DFT relaxation vs. the ones from IZA database.

Table S2. Sets of values chosen from the data visualized in Figure S7 with the best accuracy in prediction of Si-O distances and T-site volumes for experimental RRUFF spectra trained on the *full* database.

	Si-O distance		TO_4 volume	
	set-1	set-2	set-3	set-4
Sigma	100	90	60	60
Wavenumber shift (cm^{-1})	− 10	0	− 90	0
Stretching the wavenumber scale (%)	0	+ 2	0	+ 8
R^2-score (%)	53	49	42	38

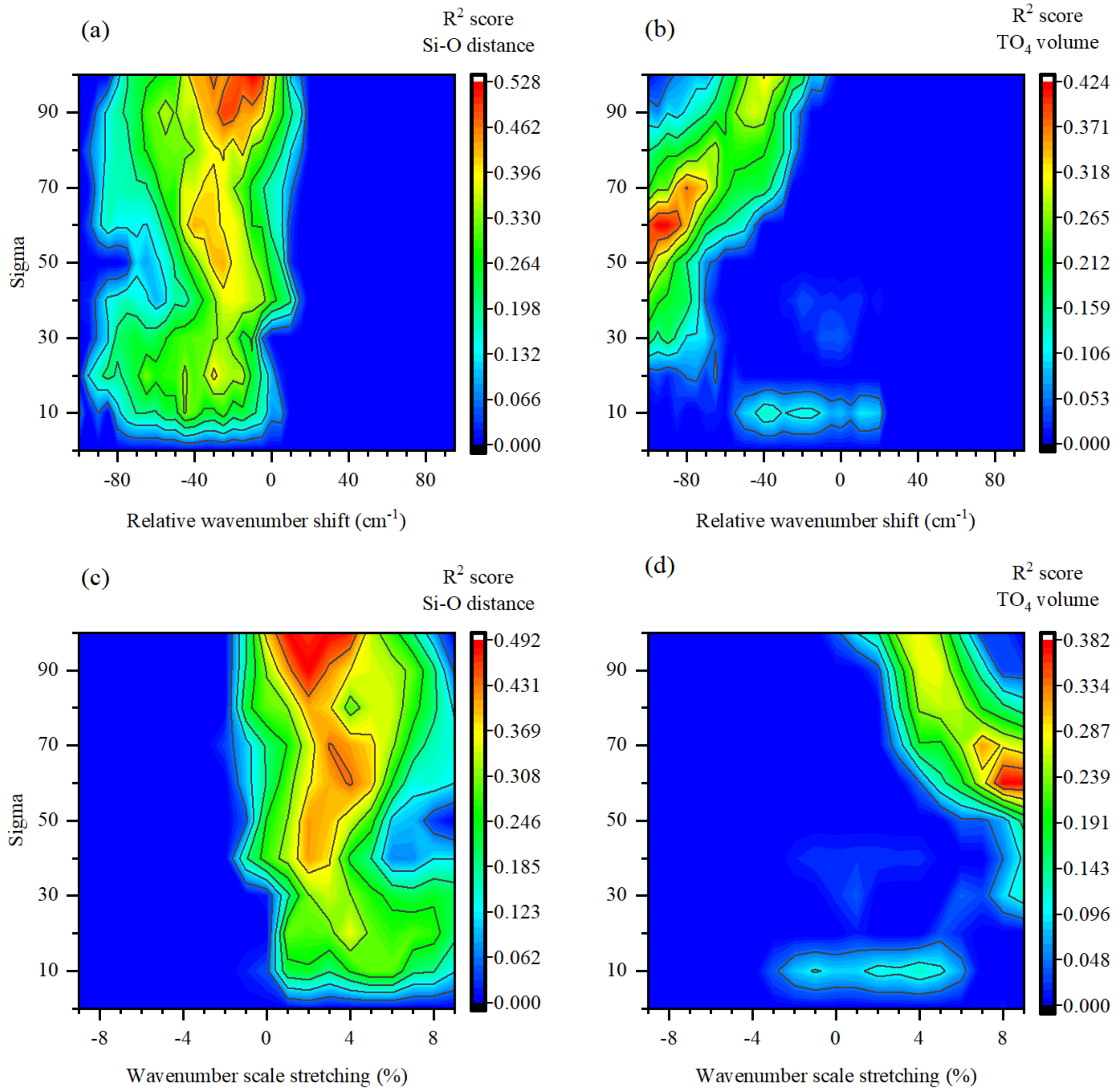

Fig. S7. Contour plot of R^2-score for Si-O distance (a, c) and T-site volume (b, d) for variation of the Gaussian function parameter sigma with wavenumber shift of theoretical spectra relative to experimental ones (a-b) and stretching the wavenumber scale of theoretical spectra (c-d).

In order to choose the best regressor, various ML learning methods were tested on the prediction of the average Si-O-Si angle using *initial* and *full* datasets (Figure S7-8). High R^2-scores were shown by Kernel Regression, Random Forest and Extra Trees. For further training, the Extra Trees method was chosen as the method with one of the highest R^2-scores.

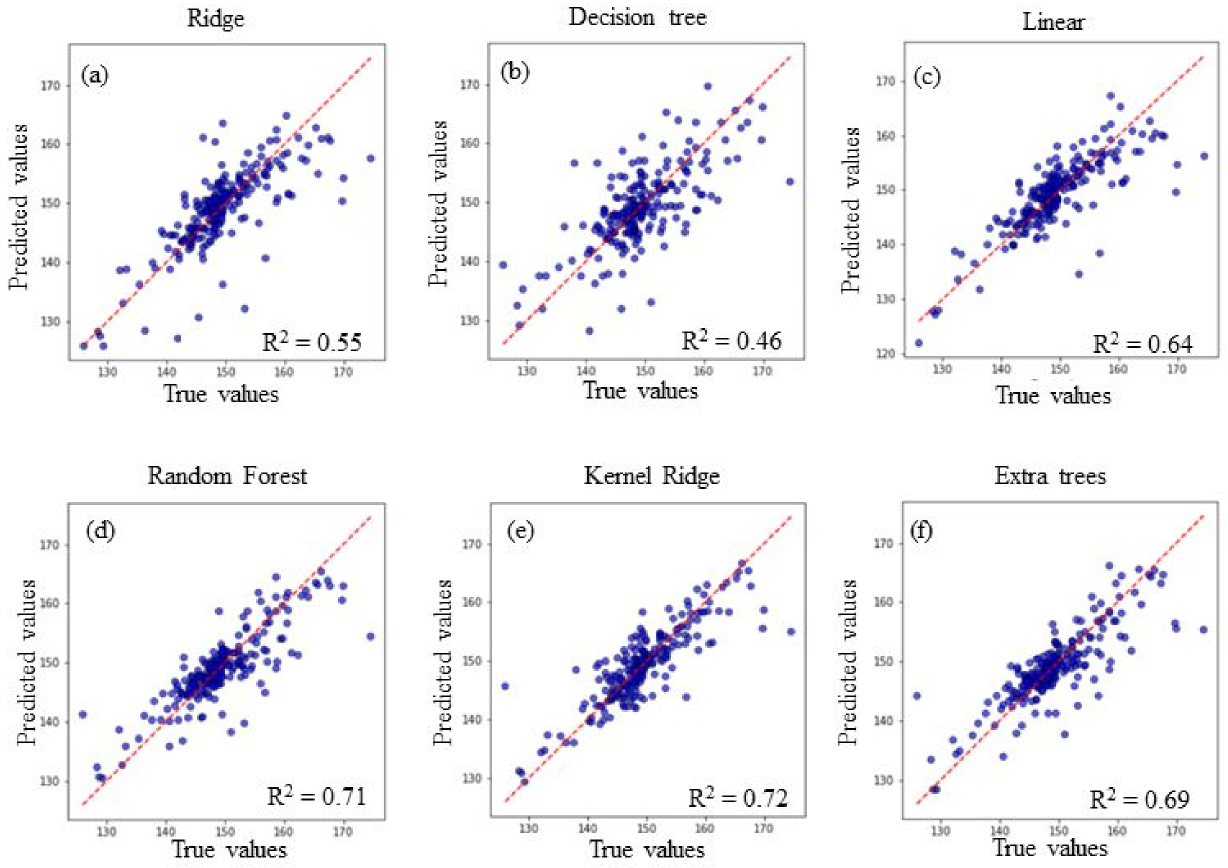

Fig. S8. Parity plot for prediction quality of averaged Si-O-Si angles on *initial* dataset using Ridge (a), Decision Tree (b), Linear (c), Random Forest (d), Kernel Ridge (e) and Extra Trees (f) methods.

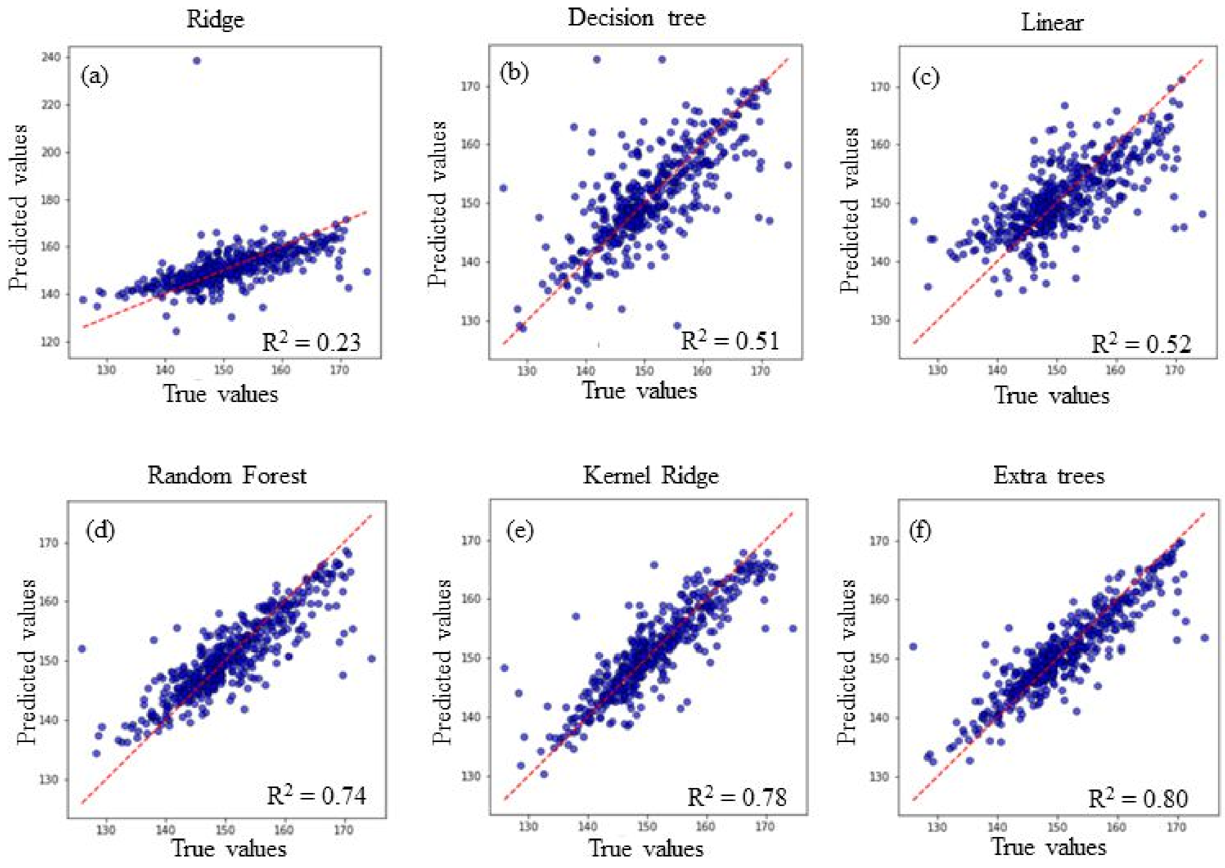

Fig. S9. Parity plot for prediction quality of averaged Si-O-Si angles on *full* dataset using Ridge (a), Decision Tree (b), Linear (c), Random Forest (d), Kernel Ridge (e) and Extra Trees (f) methods.

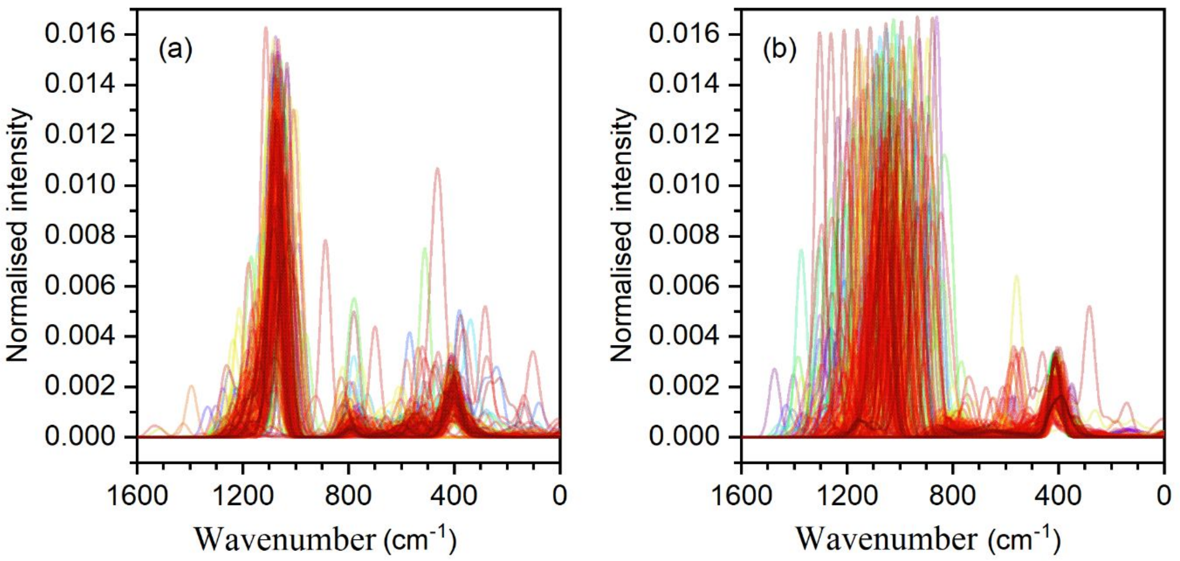

Fig. S10. Representation of the simulated IR spectra for (a) 230 types of zeolite frameworks and (b) 30 types of frameworks with distorted cell parameters.

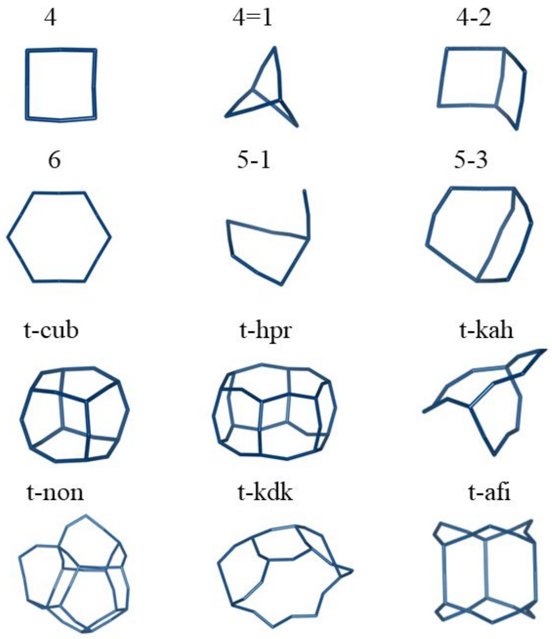

Fig. S11. Representation of some SBUs and NBUs.

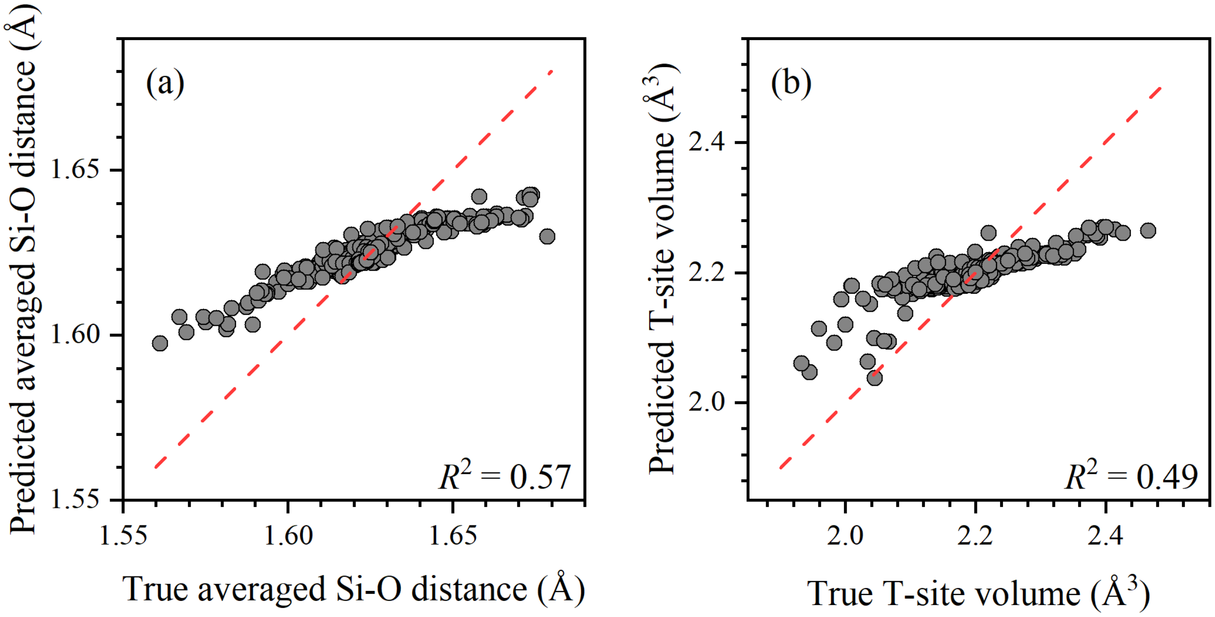

Fig. S12. Parity plot for the tetrahedron volume (a) and the average Si-O distances (b) obtained by applying Extra Trees algorithm trained on the *initial* set of 230 undistorted structures to the test set of spectra of distorted zeolites.

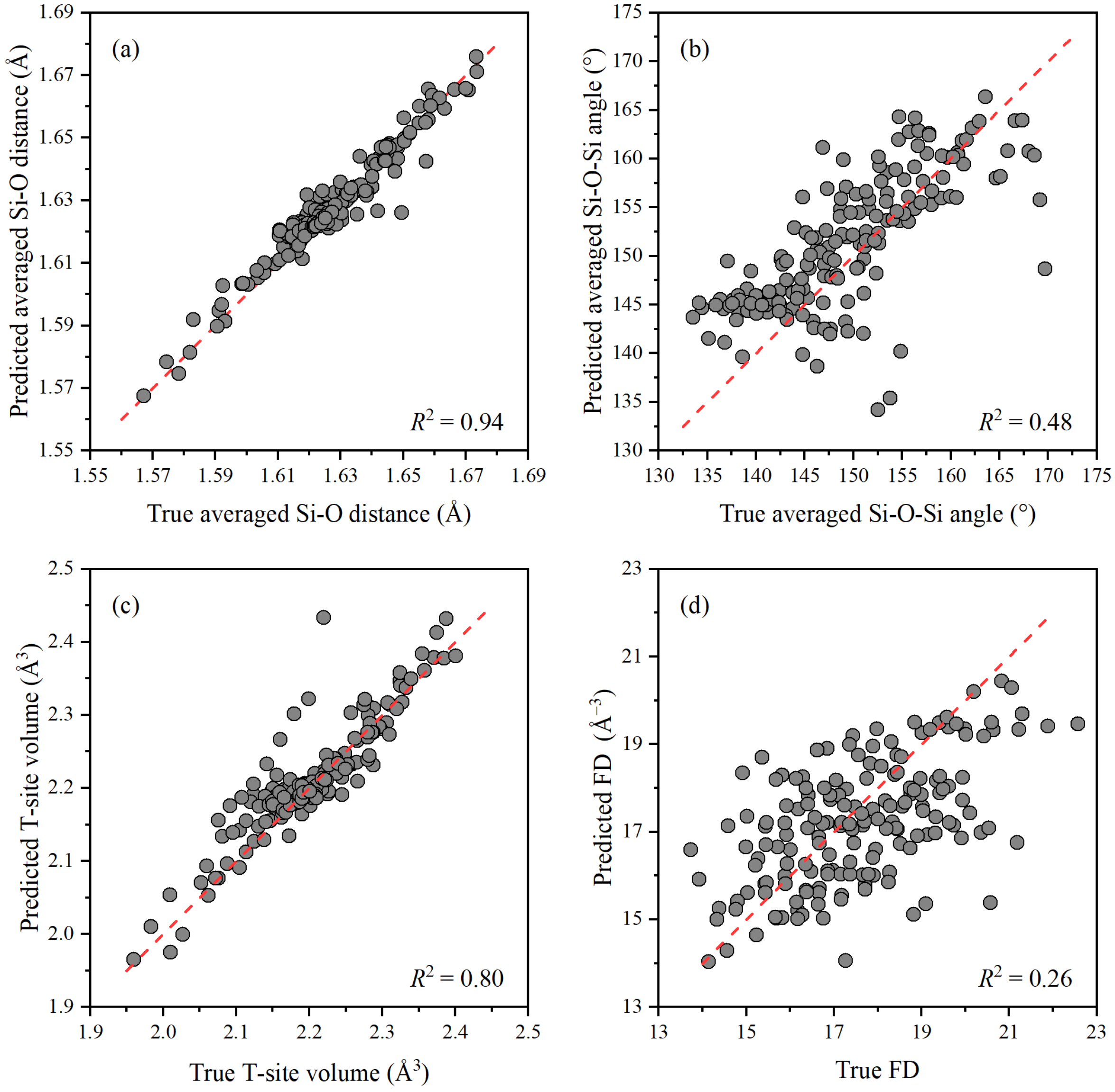

Fig. S13. Parity plot for average Si-O distances (a), average Si-O-Si angles (b), tetrahedron volume (c) and FD (d) for *mixed* training set where the test set includes the spectra of stretched structures not represented in the training set.

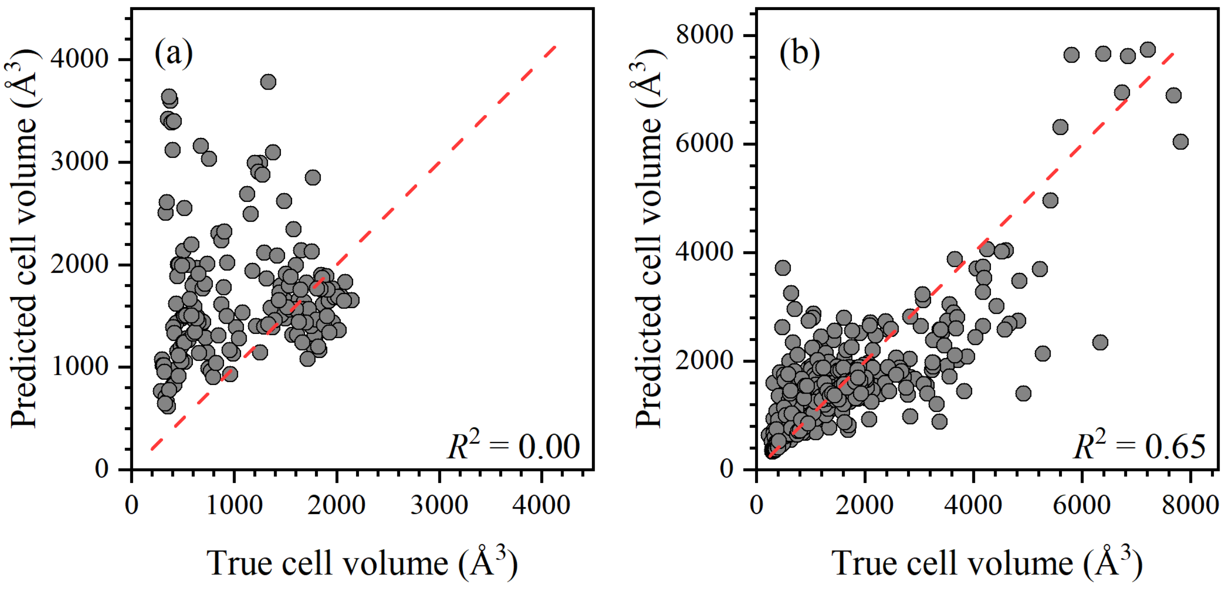

Fig. S14. Parity plot for computational cell volume given by ExtraTrees algorithm (a) trained on *mixed* training set and applied to the test set of spectra calculated for distorted structures not represented in the training set, and (b) trained on the *full* training set of all 470 simulated spectra.

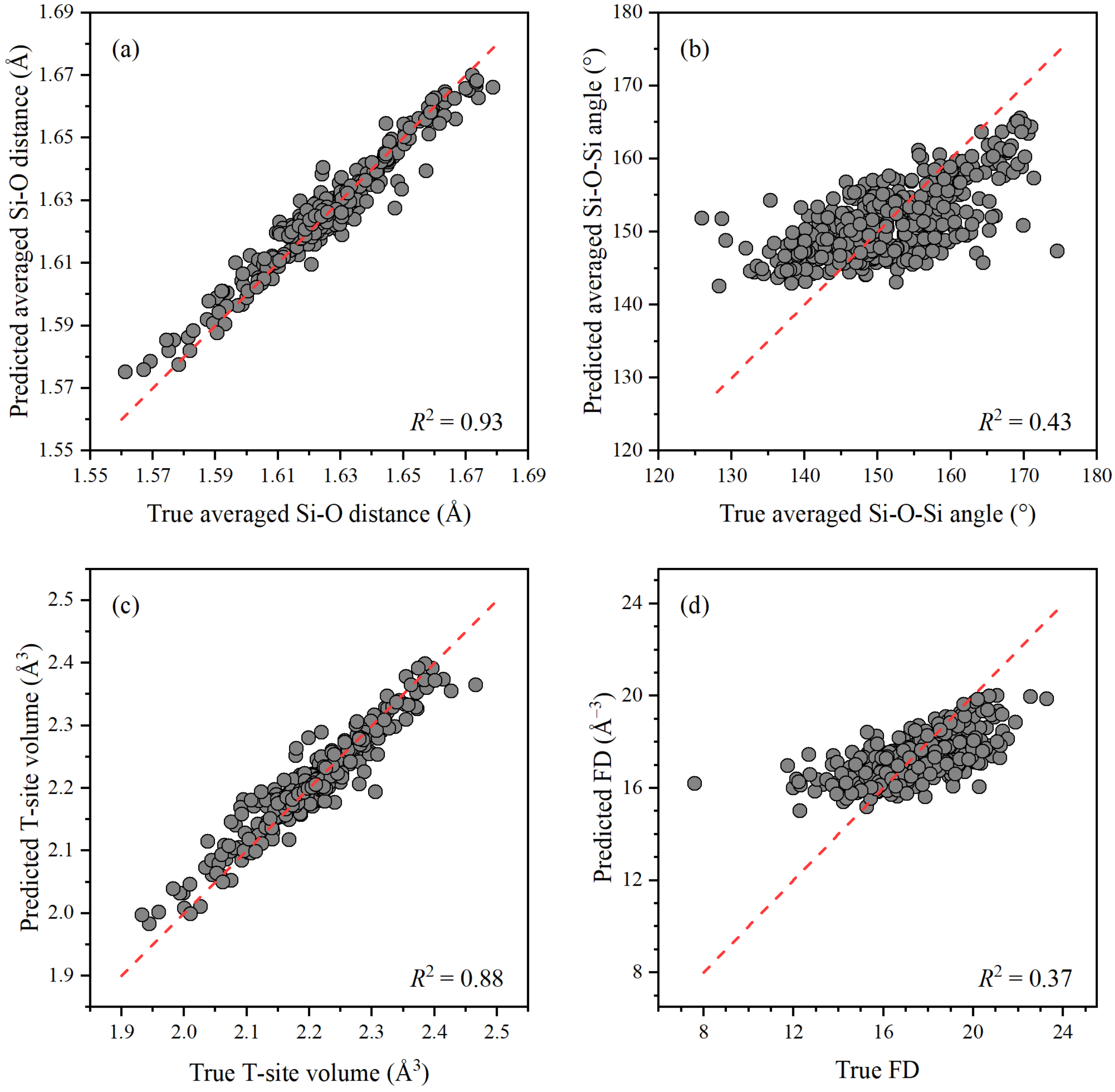

Fig. S15. Parity plot for average Si-O distances (a), average Si-O-Si angles (b), tetrahedron volume (c) and FD (d) for a *non-convoluted* dataset in which, instead of the spectrum, the prediction was carried out on a set of frequencies of the first 36 brightest vibrational modes.

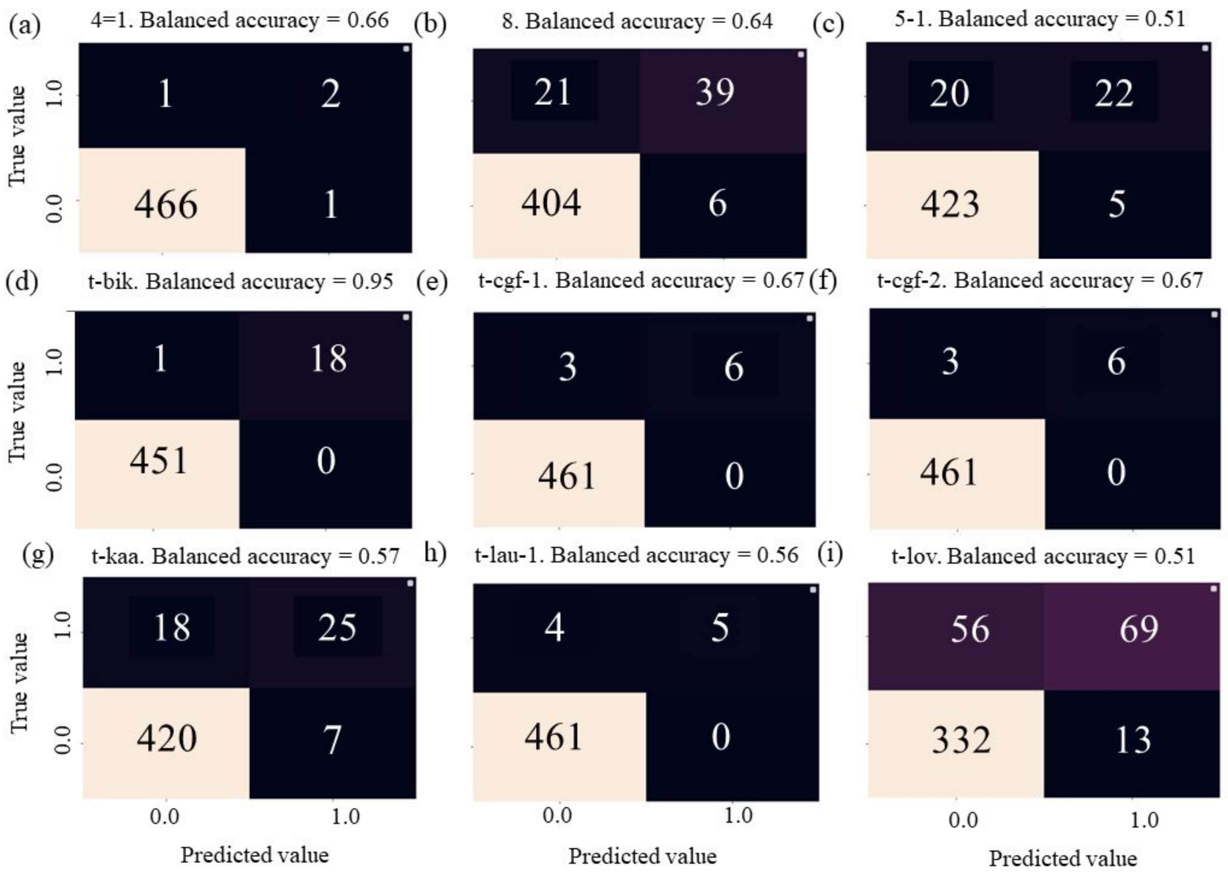

Fig. S16. Confusion matrices in solving the classification problem for SBUs 4=1 (a), 8 (b), 5-1 (c) and NBUs t-bik (d), t-cgf-1 (e), t-cgf-2 (f), t-kaa (g), t-lau-1 (h) and t-lov (i) based on *non-convoluted* dataset.

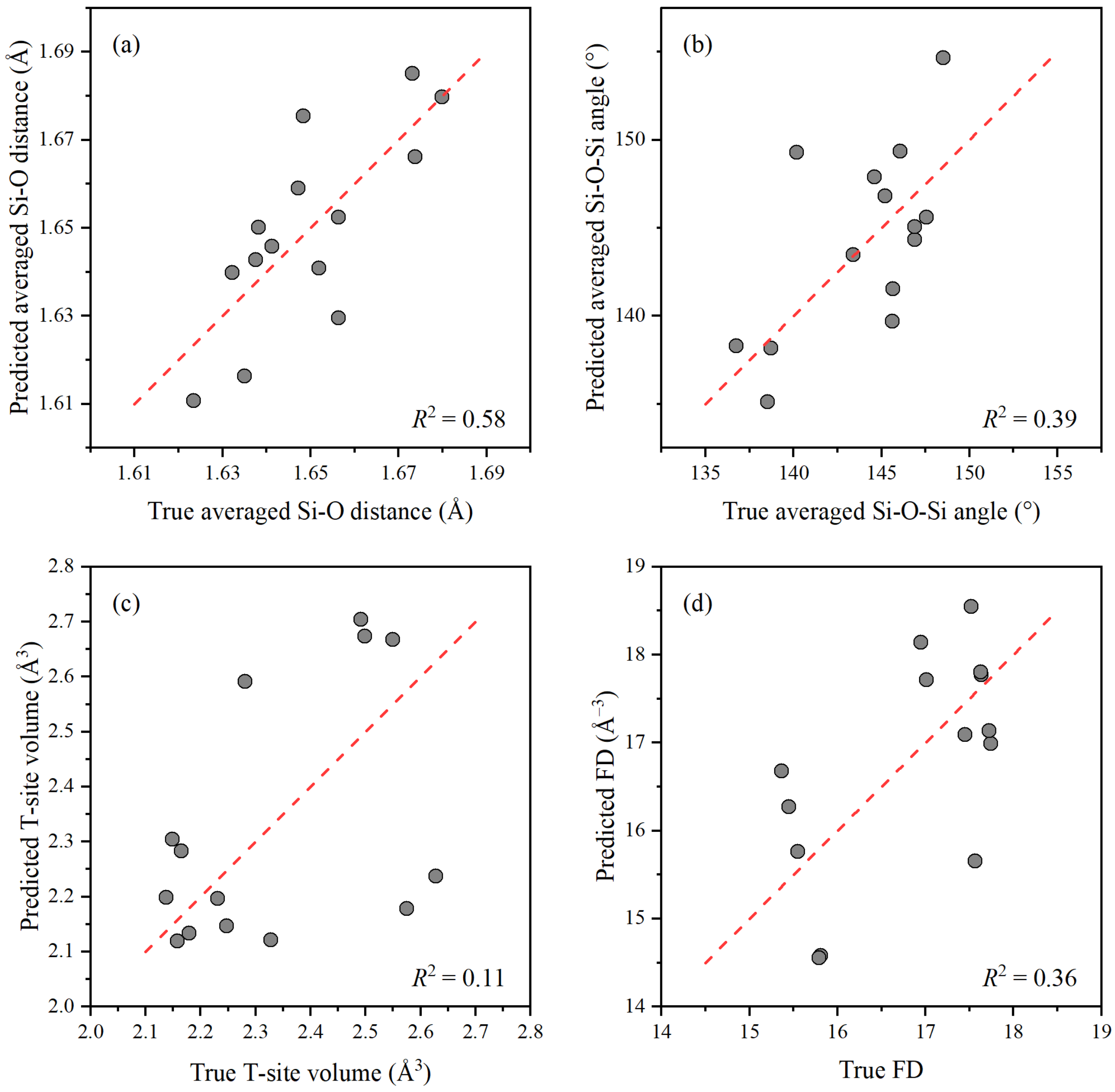

Fig. S17. Parity plot for average Si-O distances (a), average Si-O-Si angles (b), tetrahedron volume (c) and FD (d) for experimental data using ExtraTreesRegressor.

REFERENCES

1. Lafuente, B.; Downs, R. T.; Yang, H.; Stone, N., 1. The power of databases: The RRUFF project. In *Highlights in Mineralogical Crystallography*, Thomas, A.; Rosa Micaela, D., Eds. De Gruyter (O): Berlin, München, Boston, 2016; pp 1-30.
